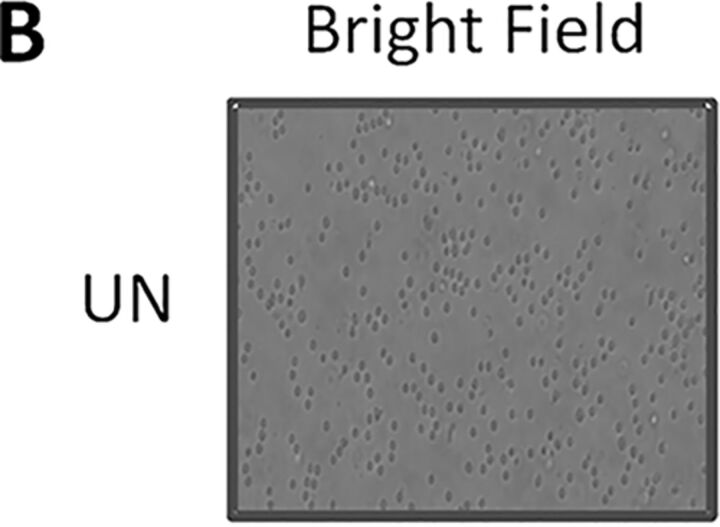# Erratum for Jha et al., “Gammaherpesvirus Infection of Human Neuronal Cells”

**DOI:** 10.1128/mBio.02981-20

**Published:** 2021-01-05

**Authors:** Hem Chandra Jha, Devan Mehta, Jie Lu, Darine El-Naccache, Sanket K. Shukla, Colleen Kovacsics, Dennis Kolson, Erle S. Robertson

**Affiliations:** a Department of Microbiology and the Tumor Virology Program, Abramson Cancer Center, Perelman School of Medicine at the University of Pennsylvania, Philadelphia, Pennsylvania, USA; b Department of Neurology, Perelman School of Medicine at the University of Pennsylvania, Philadelphia, Pennsylvania, USA

## ERRATUM

Volume 6, no. 6, e01844-15, 2015, https://doi.org/10.1128/mBio.01844-15. In Fig. 6B a duplicated image appeared as the untreated control for the Bright Field image for 5 dpi. The image should appear as shown below. There are no other changes to the text or figure legend.

**Figure:**